# Effect of Hybridization on Somatic Mutations and Genomic Rearrangements in Plants

**DOI:** 10.3390/ijms19123758

**Published:** 2018-11-27

**Authors:** Tufail Bashir, Ratnesh Chandra Mishra, Md. Mohidul Hasan, Tapan Kumar Mohanta, Hanhong Bae

**Affiliations:** 1 Department of Life sciences, Imperial College London, South Kensington SW7 2AZ, UK; t.tufail@imperial.ac.uk; 2 Department of Biotechnology, Yeungnam University, Gyeongsan, Gyeongbuk 38541, Korea; ratnesh.id@gmail.com (R.C.M.); mhasan@hstu.ac.bd (M.M.H.); 3 Natural and Medicinal Plant Research Center, University of Nizwa, Nizwa 616, Oman; nostoc.tapan@gmail.com

**Keywords:** allopolyploids, somatic mutation, transposition, hybrid plants, chromosomal rearrangements, chromosomal elimination, chromosomal expansion, centromere, homologous recombination, genome size

## Abstract

Hybridization has been routinely practiced in agriculture to enhance the crop yield. Principally, it can cause hybrid vigor where hybrid plants display increased size, biomass, fertility, and resistance to diseases, when compared to their parents. During hybridization, hybrid offspring receive a genomic shock due to mixing of distant parental genomes, which triggers a myriad of genomic rearrangements, e.g., transpositions, genome size changes, chromosomal rearrangements, and other effects on the chromatin. Recently, it has been reported that, besides genomic rearrangements, hybridization can also alter the somatic mutation rates in plants. In this review, we provide in-depth insights about hybridization triggered genomic rearrangements and somatic mutations in plants.

## 1. Introduction

In 1876, Charles Darwin described heterosis or hybrid vigor as a phenomenon, which results in enhanced crop yields by hybridizing distantly related maize varieties. Since then, hybridization in plants has been routinely practiced in agriculture to increase the productivity of crop plants [1]. Hybridization and other improved agronomic practices increased the global crop yield (from 1960s to 1990s), for example, a 132% increase in the total rice production and a 91% increase in the wheat production was seen during this period [2]. This unprecedented increase in the production of food grains has been referred to as the “green revolution” [3]. As our dependence on hybrid crops has also increased, it calls for the detailed analysis of hybridization related effects. It is important to note that hybridization is largely common in nature. Around 25% of plants and 10% of animals hybridize with at least one other species [4]. Therefore, it is necessary to study the changes that hybridization can cause within a genome—very famously referred to as a “genomic shock” by Barbara McClintock [5].

Hybridization is also inextricably linked with speciation and therefore has an important evolutionary significance. Speciation allows unification and functioning of diverged genomes from different species naturally [6]. Speciation is sometimes associated with polyploidy or whole genome duplications [7]. Duplication of the same genome is called autopolyploidy whereas allopolyploidy results from interspecific hybridization, which influences chromosomal pairing behavior, and this can consequently trigger sterility due to the production of defective gametes in hybrids [8]. To avoid infertility in allopolyploids, doubling of chromosomes occurs, which ensures that each chromosome finds a partner to pair with [9].

Besides spontaneous somatic mutations, hybridization is another potential source of genetic variations in plants. Somatic mutations act as a reservoir contributing to the genetic variability in plants. Owing to the higher mutation rates in plants (than mammals and bacteria), there is always a probability of spontaneous mutations being transmitted as heritable changes to the progeny [10,11]. In plants, any somatic mutation event can be potentially drifted towards gametes. This may also depend on the site of emergence of the reproductive organs in plants, and requires the cell that acquired a somatic mutation, by chance, to act as a progenitor of the floral organs. Certainly, acquiring such mutations may also depend on the rates of the spontaneous mutation in the plant. Previously, whole genome sequencing of *Arabidopsis* unveiled a spontaneous point mutation rate of 7 × 10^−9^ base substitutions per site per generation [12]. However, Kovalchuk and his colleagues found the somatic point mutations in *Arabidopsis* to be in the range of 10^−7^ to 10^−8^ per base per generation by using a GUS reporter based strategy.

Although, in the past, studies on mutations in hybrids, especially in *Drosophila*, have taken a lead. However, similar studies in plant hybrids still remain in infancy. Besides few recent studies involving analysis of mutation accumulation rates in *Arabidopsis* and peach hybrids, there is no information on this theme from other plant species [13,14,15,16]. Interestingly, the available studies insinuate that hybridization does affect mutations in offspring.

Consequent to the foregoing, this review also focuses on hybridization induced genomic rearrangements in plants. Scrutiny of the available literature brings forth scattered information on such events, mostly from the work done in allopolyploids.

## 2. Hybridization Affects Somatic Mutations in Plants

Although hybridization occurs frequently in nature, surprisingly, the associated mutation rates in hybrid plants are least studied. In 1935, Kostoff proposed an increase of somatic mutation frequency in inter-specific tobacco hybrids (*Nicotiana spp*.) [17]. Appearance of striped white flowers in *Nicotiana* hybrids resulted from interspecific crosses between *Nicotiana alata* (with white flowers) and *Nicotiana tabacum* (with red flowers) plants, which insinuated for the first time that mutation rates in hybrid plants could be different from parents. In this light, Kostoff proposed that conversion of a dominant red color gene to its recessive allelic form, by a mutation, could plausibly be the reason behind the striped pattern in hybrid flowers. Also, it was interesting to observe that the reversion frequencies were higher, i.e., conversion of recessive allele (flower color) to its dominant allelic form. Moreover, only the distant crosses yield the stripped flower color pattern. Thus, hybridization may raise instability in the behavior of an allele. However, the precise molecular explanation for the behavior observed by Kostoff remains obscure. Although, such results may be explained by inactivation of a floral pigmentation gene due to the transposon movement triggered by hybridization or also due to post-transcriptional gene silencing events [18,19].

Except for the studies done in *Arabidopsis* hybrids and peach [13,15], there are no other reports that provide in depth molecular insights about changes in the nature of different somatic mutation events in plants. Over 75 years back, initial studies reporting an increase in the mutation accumulation in hybrids were documented, e.g., *Drosophila* hybrids, arising from the crosses between *D. simulans* and *Drosophila melanogaster*, exhibited increased somatic mutation rates [20]. Similarly, enhanced mutation rates were also seen in *Drosophila* hybrids derived from the crosses between geographically distant strains of *D. melanogaster* [21]. Interestingly, increased mutation rates were also observed in the offspring derived from backcrosses between two races of *D. pseudoobscura* [22]. Furthermore, up to a 15-fold increase of somatic mutation events was seen in the hybrids of *D. melanogaster* males [23]. It is noteworthy that all these earlier reports on somatic mutations from flies were based on phenotypic studies that affect many traits, e.g., bristle shape, body, and eye color, etc. However, the precise molecular nature of such mutational events still remains obscure. Nonetheless, a recent study in *Arabidopsis* hybrids has provided more insights into this phenomenon. In this study, it was observed that hybridization influences somatic point mutation and homologous recombination (HR) events, in a parent of origin dependent fashion [13]. Likewise, sequencing of intraspecific *Arabidopsis* plants also revealed high point mutation and indel (insertion/deletion) mutation rates in heterozygote populations with an increased probability of the occurrence of a mutation event in the heterozygous sites. Also, a high mutation rate in genes that are involved in pathogen resistance was observed [15].

### Theories Behind Altered Mutation Rate Behavior in Hybrids—Trends Emerging from the Literature

Many theories have been proposed to explain the enhanced mutation accumulation phenomenon in hybrids [24,25,26]. Previously, it was speculated that mutator factors or suppressor alleles at multiple loci within the genome could control mutation rates in a natural population. By large, the normal tendency of natural selection is to keep the mutation rates at a minimum level, which is often disrupted by hybridization [22]. The underlying mechanisms for this may vary among different populations depending upon the genetic background or modifiers involved in hybrid individuals, which is in turn linked with parental genetic makeup [21]. Hybrids arising from geographically separated populations can presumably carry subtle differences in their suppressor alleles; say by virtue of single nucleotide polymorphisms (SNPs). In this scenario, hybridization would release ‘mutator activity’ or the ‘mutation suppression effect’ of such alleles in a hybrid genetic background, triggering a pangenomic rise of mutations. The release of the mutator activity conceivably happens due to the altered functioning of protein products—which co-function in a multimeric protein complex in hybrids—that are expressed by parental suppressor alleles. It is also believed that cytoplasmic factors regulate the activity of the mutation rates in hybrids, which explains the reason behind the differences observed in the mutation rates of reciprocal hybrids. Altogether, it is presumed that hybridization induced disruption of the co-adapted protein complexes, which are inherited from natural populations, can trigger explosive mutation accumulation [21].

An alternate hypothesis also suggests the role of local DNA based effects—that modulate local mutability’s and not the distant ones—in making hybrids mutable [15,24]. DNA sequencing has also helped in drawing correlations from intragenomic data that insinuate towards the fact that local mutation (SNPs) may accrue around the heterozygous loci [27]. Moreover, a recent study in peach bolsters the above hypothesis, further strengthening the notion that the rates of mutations may be higher in the genomic zones of heterozygosity [14]. However, by employing linkage analysis in flies, Demerec highlighted the role of global and not the local modifiers in the raised mutation rates phenomenon observed in hybrids [26]. So far, there is no convincing data supporting firmly the theory of localized heterozygosity and its association with increased mutations. It seems that the literature is more inclined towards the view of hybridization affecting mutations in a global fashion, rather than local [13,21,22,28].

## 3. Hybridization Alters Genome Size

Hybridization has the potential to substantially increase the genome size in a single generation. It can also trigger formation of new species naturally by polyploidy [29]. Hybridization is well known to alter the genome size in animals and plant species, e.g., in *Drosophila*, wallabies, and beans [30,31,32,33]. Interestingly, in the sunflower, adaptability of hybrids to their respective natural habitats has been linked with the genome size changes, and genome size increase has been believed to favor the transition of sunflower hybrids to unfavorable habitats. For instance, three homoploid *Helianthus* hybrids (*H. deserticola*, *H. anomalus*, and *H. paradoxus*) inhabit extreme environments of the desert floor, sand dunes, and salt marshes, respectively. In fact, the need of genomic redundancy to counter the effects of chromosomal rearrangement / deletion events and gametes lethality in *H. annuus* and *H. petiolaris* is plausibly the reason behind the enlargement of genome size in hybrids. This happens possibly due to the differences in chromosomal rearrangements, which result in deletions [34,35]. Moreover, in *Helianthus*, homoploid hybrids display higher (50%) nuclear DNA content than their parents. Such variability in the genome size is interesting because both hybrids and parents have the same chromosomal count and are diploids [34,36]. Interestingly, in the sunflower, frequent hybridization with much lesser fertility rates (<1%) have been reported for *H. annuus* and *H. petiolaris* parental species in the wild, and hybrid homoploids display strong crossing barriers with their parents, owing to extensive chromosomal rearrangements in hybrids [34]. Genome size variations in interspecific reciprocal hybrids of *H. annuus* and *H. petiolaris* have been attributed to the influence of nuclear–cytoplasmic interactions and a very similar phenomenon has also been observed in *Dasypyrum villosum* [37].

It is interesting to note that differences in the genome size have no bearing in general with the organismal complexity. For instance, with no apparent size differences, the genome size of diploid Poaceae ranges from 1.11 pg in *Oryza sativum* to 20.90 pg in *Secale cereale* [34]. The phenomenon of a lack of correlation between the genome size and gene numbers led to the conception of the C value paradox. There exist some adaptive explanations to answer such changes; like, for example, a small genome of invasive pines is correlated with its small seed size and high invasiveness [38,39]. It is also observed that endangered species have a larger genome size than their more common counterparts within a family [40]. Largely, the polyploidy level has been attributed to an increase in the genome size, e.g., natural polyploids within Asteraceae exhibit an increase of total DNA content as the ploidy level rises, this is also accompanied by the decrease in the DNA content of each genome in a polyploid nuclei [41]. Other mechanisms proposed for genome size changes in diploids include illegitimate recombination events and also differences arising due to the intron size variations [42,43].

Genomes may shrink by a slow process of deletion bias in small indels; they may also grow by a faster mechanism of insertion bias in large indels. Other mechanisms explaining changes in the genome size includes the DNA loss mechanism, elimination of entire chromosome, unequal crossing over, etc., as discussed later in the review. It is known that small deletions outnumber insertions of a similar size in the protein-coding sequences. Large and more frequent deletions also occur in the non-coding regions [44,45]. This deletion bias is hypothesized to happen as a result of the thermodynamics of replication slippage events in the genome, whereas insertions arise due to melting and re-replication of a previously duplicated DNA sequence, and deletions require omission of unreplicated bases. Hence, indel bias in non-coding DNA is sufficient to explain a net loss of DNA. It is also important to point that this proposed mechanism of DNA loss does not correlate with insertion or deletion events that happen outside the 1–400 bp range, because large indels will lead to a net DNA gain. This happens because large deletions may be an unlikely event; due to their influence on the gene function. While, as in the case of the insertions, its location and not its length affects the gene function [44].

### Transposition Events Play an Important Role in Bringing Genome Size Changes and Cause Genomic Restructuring in Hybrids

Mobilization of transposable elements (TE) can bring major changes in the genome architecture and biological functioning of an organism, as transposable elements constitute half of the total genome in eukaryotes. Their dynamics is thus pivotal in maintaining the genome size of a species [46]. Transposons can influence the evolutionary trajectory of an organism by altering gene function via insertion or by inducing chromosomal rearrangements. Transposons also act as a source of coding and noncoding material, which later determines genetic novelty in the form of new regulatory sequences [46]. Transposon induced chromosomal rearrangements happen when the termini from two transposons synapse together, which results in chromosomal inversions, deletions, duplications, and translocation.

Among all the TEs, class I elements, also called long terminal repeat (LTR) retrotransposons, play a decisive role in the genome size variations of angiosperms. They constitute the bulk of the plant genome, and accumulate as a result of the large scale amplification of few LTR retrotransposons [47]. The role played by retrotransposition events could be thus crucial in bringing the genome size changes in hybrids, e.g., up to a 24-fold (1330 Mb) increase of *Ty3/gypsy* retrotransposon sequence has been noticed in *Helianthus* hybrids [36]. Also, microarray based experimental observations in the past have shown the activation of En-Spm transposon in allopolyploid hybrids, and similar results were also noted in wheat experiments [33]. Other reports about proliferation of transposons in naturally growing hybrid sunflowers also bolsters the fact that hybrids may possess increased predisposition towards transposition events. Moreover, increased transposition rates have also been observed in other model organisms, e.g., in *Drosophila* and mammalian hybrids, which may lend credence to the phenomenon being more general [32,33].

DNA methylation represses transposon mobilization and if it fails to happen, it could possibly lead to major genomic alterations. Corroboratively, in the interspecific mammalian hybrids (*Macropus eugenii* × *Wallabia bicolor*), the genome wide undermethylation was linked to retroviral element amplification in centromeric heterochromatin, and chromosome remodeling. These gross chromosomal changes did not affect developmentally important genes, but reproductively, the hybrid was sterile with no spermatogenesis [33]. These reports also bolster the claim that hybridization reduces TE silencing; however, there is limited evidence linking the TEs directly with duplication events in the genome, which consequently changes the genome size. Proliferation of transposons in duplicated regions of the genome has raised some speculations about their role in duplication, but it is still not conclusive [48,49].

Taken together, numerous studies unveil that the role of TEs in genome size dynamics can be unpredictable in plants, as gymnosperms, for instance have retained the characteristics of constant genome size over millions of years, with low TE proliferation. However, other plants lineages, like *Oryza brachyantha* and *Gossypium*, show rampant TE amplification [41]. Until now, not much has been known about the molecular players that trigger dramatic amplification of retrotransposons, thereby affecting genome size in hybrids. Also, the evolutionary significance of such phenomenon remains elusive. It will be worthwhile to explore the processes affiliated with the removal of retrotransposons in a hybrid genome; the mechanisms with which they integrate into the neighboring genes. Further, it will be interesting to explore ways by which use of plant retrotransposons can help in improvement of crops, given their enormous potential as genetic tools in mapping and gene tagging [50].

## 4. Genomic Restructuring in Hybrids: The Role Played by Recombination Events in Facilitating Changes in the Architecture of the Genome

Homologous recombination (HR) occurs in all organisms. It plays a central role in the repair of DNA double stranded breaks (DSBs), which helps in genetic exchange and functions in the telomere maintenance. A recombination event between two non-identical DNA segments is known as homeologous recombination and it is often deleterious in nature [51,52,53]. Recombination-induced deletion of the repeated genomic regions leads to genomic instabilities and chromosomal rearrangements. In allopolyploids, pairing and subsequent recombination between homeologous chromosomes impedes normal progression of meiotic events, as it results in the formation of trivalents and univalents. It is also interesting to note that homeologous recombination induced genomic restructuring is quite frequent in newly formed allopolyploids as compared to the established ones. This may be further explained by the fact that in allopolyploids, progressive genomic differentiation might hamper synapsis and recombination in homeologous chromosomes, which results in diploid-like meiosis. Interestingly, the pairing homeologous 1 (*Ph1*) locus suppresses pairing of homeologous chromosomes in wheat by some elusive molecular mechanism. Subsequently, it was speculated that Ph1 screens the similarity patterns between chromosomal pairs and allows recombination only if a certain threshold similarity is achieved [54,55,56].

**Figure 1:**
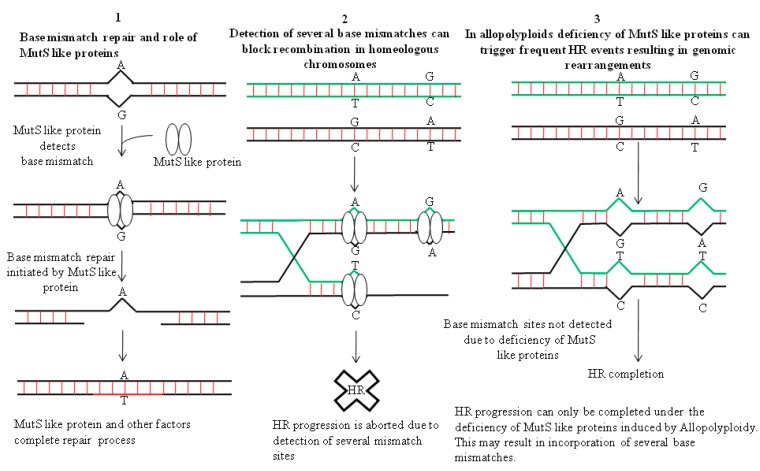
Recombination events in allopolypoids can happen frequently due to mismatch repair malfunction. (**1**) MutS like protein binds to the base mismatch site and initiates DNA repair. (**2**) Two homeologous DNA sequences can be seen which differ by virtue of some SNPs. Detection of base mismatch sites (after strand invasion) by MutS like repair machinery aborts progression of HR. (**3**) Recombination between homeologous DNA sequences/chromosomes carrying several base mismatches may proceed smoothly in allopolyploids (after strand exchange and Holliday junction formation). This can arise due to the deficiency of mismatch repair proteins resulting from their over occupancy at many mismatch sites in allopolyploids. This might trigger the presence of some mismatch zones devoid of MutS like proteins, thereby allowing completion of recombination. Based on data from [56].

**Figure 2:**
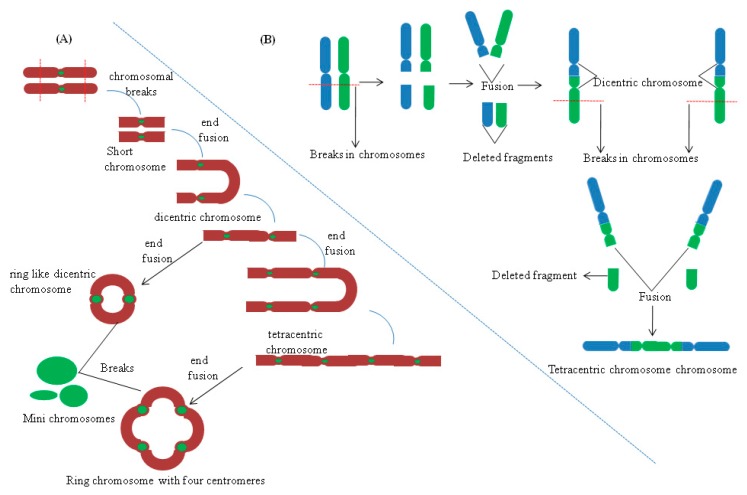
Hybridization and its effect on centromere and chromosomal stability. (**A**) Formation of ring like chromosomes and mini chromosomes with multiple centromeres. (**B**) Formation of dicentric and multicentric chromosomes in progenies of wheat rye hybrids. Based on data from [59].

In allopolyploids, formation of a Holliday junction (HJ) between homeologous chromosomes can introduce many base mismatches within complimentary /adjacent DNA strands (Figure 1). HJ is a DNA branch point formed by the covalent connections of four DNA helices by strand exchange during recombinational repair [57]. Hypothetically, the active DNA repair process should stall such recombination events in allopolyploids due to the occupancy of base mismatch sites in the genome by MutS like proteins, e.g., MSH2 [56,58]. However, due to the occurrence of a plethora of genome wide mismatch events in allopolyploids, depletion of MutS like proteins from some mismatch sites in the DNA is much more plausible. This can allow smooth progression of recombination, which otherwise would have been prevented due to active surveillance by mismatch repair machinery (Figure 1). Thus, genomic instabilities can arise due to such homeologous recombination events in allopolyploids. Additionally, genomic instabilities in allopolyploids can also trigger formation of dicentric and acentric chromosomes (without centromere), by virtue of a recombination event between two homeologous chromosomes (Figure 2). Also, intrachromosomal recombination between two tandem/adjacent, homeologous DNA fragments can initiate deletions [56].

Structural changes in the chromosomes involving loss of parental DNA and appearance of novel DNA sequences in hybrids has already been previously observed [60]. From the analysis of manmade synthetic allopolyploids, we now understand that allopolyploidization induced recombination events and transposition activation is plausibly the reason behind such genomic changes [56]. It is well recognized that the genome of allopolyploids carries considerable modifications when compared with parents. Due to unavailability of the information about original parents, predictions about the genome restructuring in allopolyploids is drawn from the inferred parental genomic information as understood from the genomic structure of their presumed diploid descendants.

## 5. Hybridization Triggers Changes in Chromosome Architecture

### 5.1. Chromosomal Elimination

Ideally, there are two copies of chromosomes in plants and animals, each derived from one parent. However, occasionally a complete set of chromosome from one parent is left out during reproduction, giving rise to the uniparental chromosomes in haploid offspring by a process known as genome elimination [61].

Chromosomal elimination events are common in interspecific hybrids where formation of haploid embryos is seen, e.g., offspring derived from *Hordeum marinum* × *H. vulgare* crosses. Also, some hybrids of Brassica species display complete chromosomal elimination [62]. Similarly, interspecific crosses between barley and its close relative, *H. bulbosum* (bulbous barley grass), produce haploids by complete elimination of the *H. bulbosum* chromosomes [63]. Uniparental elimination of chromosomes also leads to some bias in the inheritance of traits to the offspring [62,64].

In the allopolyploid, *Nicotiana tabacum*, it is believed that preferential elimination of the paternal repetitive sequences could be responsible for the observed genome downsizing effects [8]. Similarly, other studies also link elimination of repetitive DNA sequences with genome contraction as observed by whole genome sequencing and Fluorescence in situ Hybridization (FISH) analysis procedures in *Nicotiana* hybrids [65]. Further, studies done in wheat hybrids also display a loss of DNA sequences [8]. Moreover, other reports from wheat have revealed a loss of DNA sequences by drastic elimination of tandem repeats (pGclR-1) in synthetic amphiploids (derived from *T. aestivum* and *Ae.speltoides*) [66,67].

Disruption of the genome elimination process can result in the formation of individuals that have incomplete genomes. This is seen in some interspecific *Arabidopsis* hybrids from parents that have a mutant version of the CENH3 protein [68]. Genome elimination events after hybridization gives rise to many offspring that exist as haploids, along with some other plants that harbor chromosomal defects. For instance, some hybrids have an extra or truncated or a rearranged chromosome with truncations, and in some cases, these genomic rearrangements were stably heritable [68]. This shows the importance of CENH3 proteins in genome elimination and the shattering effect. CENH3 mediated genomic rearrangements in chromosomes are essentially accomplished in a parent of origin dependent fashion, as such changes were only seen in the chromosomes derived from the parent harboring the CENH3 mutation. Previously, it has also been hypothesized that centromere inactivity due to improper deposition of the centomere specific protein (CENH3) results in a decrease of centromeric CENH3 bound protein, thereby triggering chromosome elimination events in hordeum hybrids [64]. 

It has been proposed that loss of one of the two CENH3 parental protein isoforms in hordeum hybrids initiates degradation of the chromosomes that have less CENH3 bound on centromeres, which eventually forms micronuclei. Many mechanisms have also been postulated to explain uniparental chromosomal elimination, which includes: (a) Asynchronous nucleoprotein synthesis, manifesting in the loss of the lagging or retarded chromosomes [69]; (b) asynchronous cell cycle, triggering differences in the timing of mitotic events [70]; and (c) parent specific-inactivation of the centromeres [71,72]. Other hypotheses also attribute chromosomal elimination to the nuclear extrusion processes, degradation of alien chromosomes by host nucleases, and some other defects arising during cell cycle/spindle assembly; still, the precise molecular mechanism of this process remains elusive [64,73,74,75].

### 5.2. Chromosome Expansion in Hybrids

**Figure 3:**
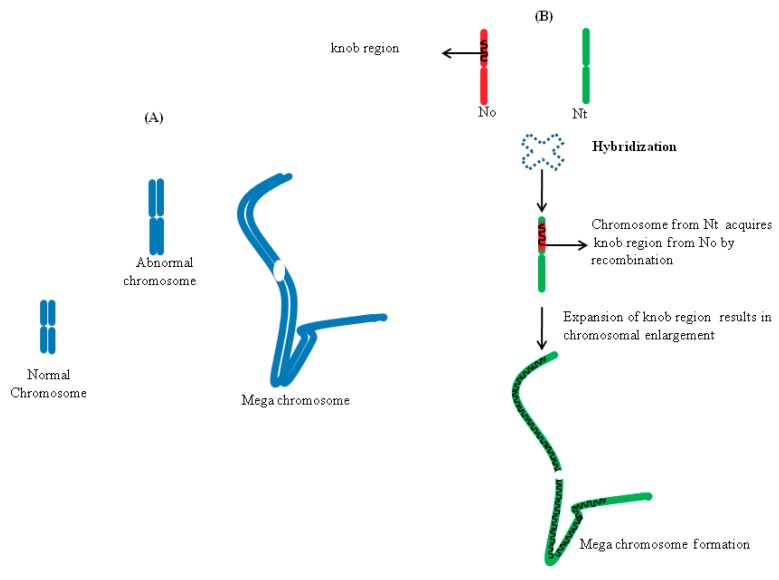
(A) Chromosomal size variations seen in *Nicotiana* allopolyploids. (B) Hybridization triggers transfer of the knob region (heterochromatinised DNA segment) via recombination from *Nicotiana tabacum* (N.t.) to *Nicotiana otophora* (N.o.) chromosomes. Subsequent expansion of the knob region results in chromosomal enlargement. Based on data from [56].

Chromosome expansion in *Nicotiana* hybrids happens due to the sudden activation of a quiescent transposon in the heterochromatinised DNA segment (called a knob), which makes such regions spontaneously unstable. This leads to the expansion and chromosomal breakage at the heterochromatin knob. Interestingly, in some cells, the magnitude of the knob expansion is so drastic that it results in formation of megachromosomes, by a 30-fold increase in the chromosomal length (Figure 3) [56]. Cells carrying such megachromosomal structures are reminiscent to Barr bodies—nonfunctional X chromosomes seen in mammalian females—due to the condensed nature of the heterochromatin. Apart from megachromosomes, the formation of abnormal chromosomes (twice the normal size) is also seen in some cells (Figure 3). It will be worthwhile to explore the nature of such genome expansions to see if there is a potential role of retrotransposon replications, which might trigger genome expansions, as seen in marsupials [33].

### 5.3. Chromosomal Rearrangements

It has been argued that chromosomal rearrangements can play an important role in the process of speciation as emergence of new species is often accompanied with structural changes in the chromosomes by means of translocations, deletions, and inversion events [76]. Many authors believe that chromosomal changes are incidental to speciation and do not drive it [77]. A better understanding of the chromosomal rearrangements, for instance, the types of translocations, their frequency of occurrence, and the chromosomal counterparts involved, can shed further light on the aspects of evolution and speciation. Many chromosomal rearrangements have also been observed in several wheat accessions growing in different parts of the world [76]. Wide hybridization is known to trigger chromosomal rearrangements and many studies have shown that wheat rye hybridization can trigger instabilities and structural changes in the chromosomes [66,78,79,80,81,82,83]. Common wheat (*T. aestivum*) is a product of natural hybridization and allopolyploidy. The most frequent chromosomal abnormality observed in wheat includes single translocation events, followed by multiple translocation or complex rearrangements, and also inversion events, in the descending degree of their frequencies of occurrence [84].

Genomic and fluorescence in situ hybridization studies on the natural allopolyploid populations of *Tragopogon miscellus* have also revealed enormous chromosomal variations. Moreover, intergenomic translocations were seen in more than 75% of the population and close to 70% of the individuals displayed aneuploidy for one or more chromosomes [85]. Partial deletion in the upper arm of the chromosome (II) has been reported in synthetic allopolyploids derived from a cross between *Arabidopsis thaliana* × *Arabidopsis lyrata* and this deleted fragment constitutes the nucleolus organizing regions (NOR2) [60]. Similarly, widespread chromosomal changes involving NOR2 have been also observed in *A*. *suecica*, which is a natural allopolyploid (*Arenosa*–*Thaliana* hybrid). Interestingly, transposition of NORs—from *A. thaliana* to *A. arenosa* chromosomes—has also been reported in these hybrids [86]. Such chromosomal reorganization events triggering changes in the number or spatial position of NORs could result due to the chromosome breaks, followed by translocation or loss of the broken fragment. Alternatively, TE initiated chromosomal breaks can also manifest in the NOR related anomalies that we see in the hybrids, as some NORs are associated with TE, e.g., NOR2 is flanked by a tangled web of TE element sequences [86].

## 6. Effects of Hybridization on Chromatin Structure and Centromere Organization

Work on interspecific hybridization from non-plant species has shown that centromeric regions remain prime targets for genomic instabilities. In marsupial hybrids, maternally derived centromeric regions remain hot sites for extensive chromosomal rearrangements, chromatin structural changes, satellite repeat, and transposable element amplification [87]. Variability in teleromeric heterochromatin and centromeric regions has been seen in wheat-rye hybrids and in their progeny. Furthermore, the presence of unstable multicentric (with many centromeres) and dicentric (with two centromeres) chromosomes was also observed in the offspring of wheat-rye hybrids, 6R/6D substitution line. Formation of stable minichromosomes was also seen in the 538 progeny of some wheat hybrid lineages [88]. In hybrids, the formation of dicentric chromosomes results due to asymmetric translocation (Figure 2). It has also been speculated that amplification of terminal chromatin arises due to non-reciprocal translocation events. Alterations by means of amplification or reduction of subtelomeric repeated sequences (Spelt1 and Spelt52) have been observed in the amphiploids of *Aegilops* and *Triticum* [89]. In wheat rye hybrids progeny, 195 cases of translocations events between wheat/rye chromosomes were seen from 785 cytological samples. Predominantly, most of the translocations (188) were of the centric break fusion type. However, formation of dicentric and multicentric chromosomes has also been seen, but their frequency of occurrence is quite low.

## 7. Conclusions and Outlook

Hybridization has the potential to bring genetic diversity, increase the vigor in progeny, and it may also reduce the fitness of the individual. Genomic rearrangements seen in hybrids (Figure 2) by virtue of chromosomal breaks have been extensively studied in wheat hybrids and other plant species. However, it remains obscure how such breaks happen in the chromosomes, which eventually triggers inversion, deletion, and translocation. Furthermore, it is interesting to explore if there exists any bias in chromosomes to undergo such effects in allopolyploids, or whether it is a chance event. The regions of the chromosomes that are amenable to various chromosomal rearrangements in hybrids needs further resolution to specifically identify the DNA sequences that are involved in such rearrangement events. This in turn will provide in depth clarity and will enhance our understanding about the elusive nature of chromosomal rearrangement processes, which were previously examined by using C banding/karyotype techniques. Other than genomic rearrangements, wonderful work done in *Helianthus*, revealing genome size variations in different hybrids makes it intriguing to understand whether such changes can also happen in reciprocal hybrids. It should be possible to address this by using next generation sequencing to get in-depth insights, or with flow cytometric observations of the DAPI stained nuclei, to get some sneak peeks into the genome size variations.

Recent literature convincingly points out the fact that like *Drosophila* hybrids, hybrid plants also show altered mutation rates [13]. In this context, it will be interesting to study if the hybrid nature of protein isoforms or DNA polymerase can alter the fidelity of DNA replication, which might result in the changes observed in the mutation accumulation behavior of hybrids [13]. Lastly, we know much about hybridization and to an extent the genomic modification it brings, but least about the involved molecular mechanisms. Now, it is high time to start focusing on this lagging aspect of hybridization related studies, which certainly would accelerate both the basic and applied research in this field.

## References

[1] Darwin C.R. (1876). The Effects of Cross and Self Fertilization in the Vegetable Kingdom.

[2] Duvick D.N., Coors J.G., Pandey S. (1999). Heterosis: Feeding people and protecting natural resources. Genetics and Exploitation of Heterosis in Crops.

[3] Khush G.S. (2001). Green revolution: The way forward. Nat. Rev. Genet..

[4] Mallet J. (2007). Hybrid speciation. Nature.

[5] McClintock B. (1984). The significance of responses of the genome to challenge. Science.

[6] Chen Z.J. (2010). Molecular mechanisms of polyploidy and hybrid vigor. Trends Plant Sci..

[7] Del Pozo J.C., Ramirez-Parra E. (2015). Whole genome duplications in plants: An overview from Arabidopsis. J. Exp. Bot..

[8] Shaked H., Kashkush K., Ozkan H., Feldman M., Levy A.A. (2001). Sequence elimination and cytosine methylation are rapid and reproducible responses of the genome to wide hybridization and allopolyploidy in wheat. Plant Cell.

[9] Vitte C., Bennetzen J.L. (2006). Analysis of retrotransposon structural diversity uncovers properties and propensities in angiosperm genome evolution. Proc. Natl. Acad. Sci. USA.

[10] Baake E., Gabriel W. (1999). Biological evolution through mutation, selection and drift: An introductory review. Annu. Rev. Comp. Phys..

[11] Kovalchuk I., Kovalchuk O., Hohn B. (2000). Genome-wide variation of the somatic mutation frequency in transgenic plants. EMBO J..

[12] Ossowski S., Schneeberger K., Lucas-Lledo J.I., Warthmann N., Clark R.M., Shaw R.G., Weigel D., Lynch M. (2010). The rate and molecular spectrum of spontaneous mutations in Arabidopsis thaliana. Science.

[13] Bashir T., Sailer C., Gerber F., Loganathan N., Bhoopalan H., Eichenberger C., Grossniklaus U., Baskar R. (2014). Hybridization alters spontaneous mutation rates in a parent-of-origin-dependent fashion in Arabidopsis. Plant Physiol..

[14] Xie Z., Wang L., Wang L., Wang Z., Lu Z., Tian D., Yang S., Hurst L.D. (2016). Mutation rate analysis via parent-progeny sequencing of the perennial peach. I. A low rate in woody perennials and a higher mutagenicity in hybrids. Proc. Biol. Sci..

[15] Yang S., Wang L., Huang J., Zhang X., Yuan Y., Chen J.Q., Hurst L.D., Tian D. (2015). Parent-progeny sequencing indicates higher mutation rates in heterozygotes. Nature.

[16] Wang L., Zhang Y., Qin C., Tian D., Yang S., Hurst L.D. (2016). Mutation rate analysis via parent-progeny sequencing of the perennial peach. II. No evidence for recombination-associated mutation. Proc. Biol. Sci..

[17] Kostoff D. (1935). On the increase of mutation frequency following interspecific hybridization in Nicotiana. Curr. Sci..

[18] Napoli C., Lemieux C., Jorgensen R. (1990). Introduction of a Chimeric Chalcone Synthase Gene into Petunia Results in Reversible Co-Suppression of Homologous Genes in trans. Plant Cell.

[19] Gerats A.G., Huits H., Vrijlandt E., Marana C., Souer E., Beld M. (1990). Molecular characterization of a nonautonomous transposable element (dTph1) of petunia. Plant Cell.

[20] Belgovsky M.L. (1937). A comparison of the frequency of induced mutations in D. simulans and in its hybrid with D. melanogaster. Genetica.

[21] Thompson J.N., Woodruff R.C. (1980). Increased mutation in crosses between geographically separated strains of Drosophila melanogaster. Proc. Natl. Acad. Sci. USA.

[22] Sturtevant A.H. (1939). High Mutation Frequency Induced by Hybridization. Proc. Natl. Acad. Sci. USA.

[23] Simmons M.J., Johnson N.A., Fahey T.M., Nellett S.M., Raymond J.D. (1980). High mutability in male hybrids of Drosophila melanogaster. Genetics.

[24] Amos W. (2010). Heterozygosity and mutation rate: Evidence for an interaction and its implications: The potential for meiotic gene conversions to influence both mutation rate and distribution. Bioessays.

[25] Comai L., Madlung A., Josefsson C., Tyagi A. (2003). Do the different parental ‘heteromes’ cause genomic shock in newly formed allopolyploids?. Philos. Trans. R. Soc. Lond. B Biol. Sci..

[26] Demerec M. (1929). Changes in the Rate of Mutability of the Mutable Miniature Gene of Drosophila Virilis. Proc. Natl. Acad. Sci. USA.

[27] Amos W. (2010). Even small SNP clusters are non-randomly distributed: Is this evidence of mutational non-independence?. Proc. Biol. Sci..

[28] Woodruff R.C., Thompson J.N., Lyman R.F. (1979). Intraspecific hybridisation and the release of mutator activity. Nature.

[29] Bennetzen J.L., Ma J., Devos K.M. (2005). Mechanisms of recent genome size variation in flowering plants. Ann. Bot..

[30] Rogers S.O., Bendich A.J. (1987). Heritability and Variability in Ribosomal RNA Genes of Vicia faba. Genetics.

[31] Petrov D.A., Schutzman J.L., Hartl D.L., Lozovskaya E.R. (1995). Diverse transposable elements are mobilized in hybrid dysgenesis in Drosophila virilis. Proc. Natl. Acad. Sci. USA.

[32] Labrador M., Farre M., Utzet F., Fontdevila A. (1999). Interspecific hybridization increases transposition rates of Osvaldo. Mol. Biol. Evol..

[33] O’Neill R.J., O’Neill M.J., Graves J.A. (1998). Undermethylation associated with retroelement activation and chromosome remodelling in an interspecific mammalian hybrid. Nature.

[34] Baack E.J., Whitney K.D., Rieseberg L.H. (2005). Hybridization and genome size evolution: Timing and magnitude of nuclear DNA content increases in Helianthus homoploid hybrid species. New Phytol..

[35] Burke J.M., Lai Z., Salmaso M., Nakazato T., Tang S., Heesacker A., Knapp S.J., Rieseberg L.H. (2004). Comparative mapping and rapid karyotypic evolution in the genus helianthus. Genetics.

[36] Ungerer M.C., Strakosh S.C., Zhen Y. (2006). Genome expansion in three hybrid sunflower species is associated with retrotransposon proliferation. Curr. Biol..

[37] Caceres M.E., De Pace C., Mugnozza G.T.S., Kotsonis P., Ceccarelli M., Cionini P.G. (1998). Genome size variations within Dasypyrum villosum: Correlations with chromosomal traits, environmental factors and plant phenotypic characteristics and behaviour in reproduction. Theor. Appl. Genet..

[38] Thomas C.A. (1971). The genetic organization of chromosomes. Ann. Rev. Genet..

[39] Grotkopp E., Rejmanek M., Sanderson M.J., Rost T.L. (2004). Evolution of genome size in pines (Pinus) and its life-history correlates: Supertree analyses. Evolution.

[40] Vinogradov A.E. (2003). Selfish DNA is maladaptive: Evidence from the plant Red List. Trends Genet..

[41] Leitch I., Bennett M. (2004). Genome downsizing in polyploid plants. Biol. J. Linn. Soc..

[42] Petrov D.A. (2001). Evolution of genome size: New approaches to an old problem. Trends Genet..

[43] Bennetzen J.L. (2002). Mechanisms and rates of genome expansion and contraction in flowering plants. Genetica.

[44] Gregory T.R. (2004). Insertion-deletion biases and the evolution of genome size. Gene.

[45] Petrov D.A. (2002). Mutational equilibrium model of genome size evolution. Theor. Popul. Biol..

[46] Feschotte C., Pritham E.J. (2007). DNA transposons and the evolution of eukaryotic genomes. Ann. Rev Genet..

[47] Bennetzen J.L., Wang H. (2014). The contributions of transposable elements to the structure, function, and evolution of plant genomes. Ann. Rev. Plant Biol..

[48] Hazzouri K.M., Mohajer A., Dejak S.I., Otto S.P., Wright S.I. (2008). Contrasting patterns of transposable-element insertion polymorphism and nucleotide diversity in autotetraploid and allotetraploid Arabidopsis species. Genetics.

[49] Hughes A.L., Friedman R., Ekollu V., Rose J.R. (2003). Non-random association of transposable elements with duplicated genomic blocks in Arabidopsis thaliana. Mol. Phylogenet. Evol..

[50] Kumar A., Bennetzen J.L. (1999). Plant retrotransposons. Ann. Rev. Genet..

[51] Li L., Santerre-Ayotte S., Boivin E.B., Jean M., Belzile F. (2004). A novel reporter for intrachromosomal homoeologous recombination in Arabidopsis thaliana. Plant J..

[52] Sung P., Klein H. (2006). Mechanism of homologous recombination: Mediators and helicases take on regulatory functions. Nat. Rev. Mol. Cell Biol..

[53] Renkawitz J., Lademann C.A., Jentsch S. (2014). Mechanisms and principles of homology search during recombination. Nat. Rev. Mol. Cell Biol..

[54] Luo M.C., Dubcovsky J., Dvorak J. (1996). Recognition of homeology by the wheat Ph1 locus. Genetics.

[55] Moore G. (1998). To pair or not to pair: Chromosome pairing and evolution. Curr. Opin. Plant Biol..

[56] Comai L. (2000). Genetic and epigenetic interactions in allopolyploid plants. Plant Mol. Biol..

[57] Lilley D.M.J. (2017). Holliday junction-resolving enzymes-structures and mechanisms. FEBS Lett..

[58] Culligan K.M., Hays J.B. (2000). Arabidopsis MutS homologs-AtMSH2, AtMSH3, AtMSH6, and a novel AtMSH7-form three distinct protein heterodimers with different specificities for mismatched DNA. Plant Cell.

[59] Fu S., Lv Z., Guo X., Zhang X., Han F. (2013). Alteration of terminal heterochromatin and chromosome rearrangements in derivatives of wheat-rye hybrids. J. Genet. Genomics.

[60] Beaulieu J., Jean M., Belzile F. (2009). The allotetraploid Arabidopsis thaliana-Arabidopsis lyrata subsp. petraea as an alternative model system for the study of polyploidy in plants. Mol. Genet. Genomics.

[61] Ravi M., Chan S.W. (2010). Haploid plants produced by centromere-mediated genome elimination. Nature.

[62] Tonosaki K., Osabe K., Kawanabe T., Fujimoto R. (2016). The importance of reproductive barriers and the effect of allopolyploidization on crop breeding. Breed. Sci..

[63] Ho K.M., Kasha K.J. (1975). Genetic Control of Chromosome Elimination during Haploid Formation in Barley. Genetics.

[64] Sanei M., Pickering R., Kumke K., Nasuda S., Houben A. (2011). Loss of centromeric histone H3 (CENH3) from centromeres precedes uniparental chromosome elimination in interspecific barley hybrids. Proc. Natl. Acad. Sci. USA.

[65] Renny-Byfield S., Kovarik A., Chester M., Nichols R.A., Macas J., Novak P., Leitch A.R. (2012). Independent, rapid and targeted loss of highly repetitive DNA in natural and synthetic allopolyploids of Nicotiana tabacum. PLoS ONE.

[66] Tang Z., Li M., Chen L., Wang Y., Ren Z., Fu S. (2014). New types of wheat chromosomal structural variations in derivatives of wheat-rye hybrids. PLoS ONE.

[67] Han F., Fedak G., Guo W., Liu B. (2005). Rapid and repeatable elimination of a parental genome-specific DNA repeat (pGc1R-1a) in newly synthesized wheat allopolyploids. Genetics.

[68] Tan E.H., Henry I.M., Ravi M., Bradnam K.R., Mandakova T., Marimuthu M.P., Korf I., Lysak M.A., Comai L., Chan S.W. (2015). Catastrophic chromosomal restructuring during genome elimination in plants. Elife.

[69] Bennett M.D., Finch R.A., Barclay I.R. (1976). The time rate and mechanism of chromosome elimination in Hordeum hybrids. Chromosoma.

[70] Gupta S.B. (1969). Duration of mitotic cycle and regulation of DNA replication in Nicotiana plumbaginifolia and a hybrid derivative of N. tabacum showing chromosome instability. Can. J. Genet. Cytol..

[71] Finch R.A. (1983). Tissue-specific elimination of alternative whole parental genomes in one barley hybrid. Chromosoma.

[72] Kim N.S., Armstrong K.C., Fedak G., Ho K., Park N.I. (2002). A microsatellite sequence from the rice blast fungus (Magnaporthe grisea) distinguishes between the centromeres of Hordeum vulgare and H. bulbosum in hybrid plants. Genome.

[73] Gernand D., Rutten T., Pickering R., Houben A. (2006). Elimination of chromosomes in Hordeum vulgare x H. bulbosum crosses at mitosis and interphase involves micronucleus formation and progressive heterochromatinization. Cytogenet. Genome Res..

[74] Ishii T., Ueda T., Tanaka H., Tsujimoto H. (2010). Chromosome elimination by wide hybridization between Triticeae or oat plant and pearl millet: Pearl millet chromosome dynamics in hybrid embryo cells. Chromosome Res..

[75] Linde-Laursen I., von Bothmer R. (1988). Elimination and duplication of particular Hordeum vulgare chromosomes in aneuploid interspecific Hordeum hybrids. TAG. Theor. Appl. Genet..

[76] Badaeva E.D., Dedkova O.S., Gay G., Pukhalskyi V.A., Zelenin A.V., Bernard S., Bernard M. (2007). Chromosomal rearrangements in wheat: Their types and distribution. Genome.

[77] Raskina O., Barber J.C., Nevo E., Belyayev A. (2008). Repetitive DNA and chromosomal rearrangements: Speciation-related events in plant genomes. Cytogenet. Genome Res..

[78] Alkhimova A.G., Heslop-Harrison J.S., Shchapova A.I., Vershinin A.V. (1999). Rye chromosome variability in wheat-rye addition and substitution lines. Chromosome Res..

[79] Appels R., Gustafson J.P., May C.E. (1982). Structural variation in the heterochromatin of rye chromosomes in triticales. Theor. Appl. Genet..

[80] Bento M., Gustafson P., Viegas W., Silva M. (2010). Genome merger: From sequence rearrangements in triticale to their elimination in wheat-rye addition lines. Theor. Appl. Genet..

[81] Lapitan N.L., Sears R.G., Gill B.S. (1984). Translocations and other karyotypic structural changes in wheat x rye hybrids regenerated from tissue culture. TAG. Theor. Appl. Genet..

[82] Szakacs E., Molnar-Lang M. (2010). Molecular cytogenetic evaluation of chromosome instability in Triticum aestivum-Secale cereale disomic addition lines. J. Appl. Genet..

[83] Tang Z.X., Fu S.L., Ren Z.L., Zhou J.P., Yan B.J., Zhang H.Q. (2008). Variations of tandem repeat, regulatory element, and promoter regions revealed by wheat-rye amphiploids. Genome.

[84] Kawahara T. (1997). Screening of spontaneous translocations in cultivated emmer wheat. Wheat Inf. Serv..

[85] Chester M., Gallagher J.P., Symonds V.V., Cruz da Silva A.V., Mavrodiev E.V., Leitch A.R., Soltis P.S., Soltis D.E. (2012). Extensive chromosomal variation in a recently formed natural allopolyploid species, Tragopogon miscellus (Asteraceae). Proc. Natl. Acad. Sci. USA.

[86] Pontes O., Neves N., Silva M., Lewis M.S., Madlung A., Comai L., Viegas W., Pikaard C.S. (2004). Chromosomal locus rearrangements are a rapid response to formation of the allotetraploid Arabidopsis suecica genome. Proc. Natl. Acad. Sci. USA.

[87] Metcalfe C.J., Bulazel K.V., Ferreri G.C., Schroeder-Reiter E., Wanner G., Rens W., Obergfell C., Eldridge M.D., O’Neill R.J. (2007). Genomic instability within centromeres of interspecific marsupial hybrids. Genetics.

[88] Fu S., Yang M., Fei Y., Tan F., Ren Z., Yan B., Zhang H., Tang Z. (2013). Alterations and abnormal mitosis of wheat chromosomes induced by wheat-rye monosomic addition lines. PLoS ONE.

[89] Salina E.A., Numerova O.M., Ozkan H., Feldman M. (2004). Alterations in subtelomeric tandem repeats during early stages of allopolyploidy in wheat. Genome.

